# The Expression of lncRNAs EVADR and LUESCC in Colorectal Tumor Tissues and Their Association With the CRC Risk

**DOI:** 10.1002/cnr2.70232

**Published:** 2025-07-07

**Authors:** Mozhgan Ahmadzadeh, Kamal Shahamiri, Mohammad Raeisi, Negar Jafari, Shaghayegh Kamian, Mahsa Ejlalidiz, Niloufar Sadat Kalaki

**Affiliations:** ^1^ Department of Cellular and Molecular Biology Faculty of Biological Sciences, Kharazmi University Tehran Iran; ^2^ Cellular and Molecular Research Center Basic Health Sciences Institute, Shahrekord University of Medical Sciences Shahrekord Iran; ^3^ Clinical Research Developmental Unit Hajar Hospital, Shahrekord University of Medical Sciences Shahrekord Iran; ^4^ Department of Cardiology Urmia University of Medical Sciences Urmia Iran; ^5^ Department of Radiation Oncology Imam Hossein Hospital, School of Medicine, Shahid Beheshti University of Medical Sciences Tehran Iran; ^6^ Medical Student Research Committee School of Medicine, Shahid Beheshti University of Medical Sciences Tehran Iran; ^7^ Student Research Committee School of Medicine, Shahid Beheshti University of Medical Sciences Tehran Iran; ^8^ Department of Medical Genetics Shahid Beheshti University of Medical Sciences Tehran Iran

**Keywords:** bioinformatics, colorectal cancer, EVADR, long non‐coding RNAs, LUESCC

## Abstract

**Background:**

Colorectal cancer (CRC) is a prevalent form of cancer globally and ranks as the second most common cause of cancer‐related deaths. Long non‐coding RNAs (lncRNAs) are regulatory RNAs that influence gene expression. EVADR and LUESCC are two novel lncRNAs specifically expressed in tumors of glandular origin, such as the colon.

**Aims:**

This study aimed to investigate the expression of EVADR and LUESCC in colorectal tumor tissues and evaluate their potential as diagnostic and prognostic biomarkers in CRC.

**Methods:**

Fifty cases of colorectal tumor tissues, formalin‐fixed, paraffin‐embedded (FFPE) from individuals with sporadic CRC, referred from the Pathology Department of Imam Hossein Hospital in Tehran, Iran, were analyzed. The expression patterns of LUESCC and EVADR lncRNAs in CRC patients were examined.

**Results:**

The study reveals upregulation of LUESCC and EVADR lncRNAs in colorectal cancer (CRC) patients compared to normal tissues, with fold changes of 3.52 (*p* < 0.001) for LUESCC and 3.08 (*p* < 0.001) for EVADR. ROC curve analysis indicates an area under the curve (AUC) of 0.75 for LUESCC and 0.86 for EVADR, suggesting strong diagnostic potential. Additionally, differential expression analysis shows correlations between lncRNA levels and tumor differentiation grades.

**Conclusion:**

This study highlights the potential of LUESCC and EVADR lncRNAs as biomarkers for CRC diagnosis and prognosis. However, limitations include a small sample size that may affect the generalizability of the findings and a lack of functional assays to elucidate their roles in tumor biology. Further research is needed to validate these findings and explore the underlying mechanisms.

**Abbreviations**
CRC: colorectal cancer
EMT: epithelial‐mesenchymal transition
ESCC: esophageal squamous cell carcinoma

*F. nucleatum*

:
*Fusobacterium nucleatum*

FFPE: formalin‐fixed, paraffin‐embedded
FOB: fecal occult blood
KLF4: Krüppel‐like factor 4
lncRNA: long non‐coding RNAs
ncRNAs: non‐coding RNAs
SBMU: Shahid Beheshti University of Medical Sciences
TCGA: The Cancer Genome Atlas

## 1 Introduction

Colorectal cancer (CRC) is a prevalent form of cancer that significantly impacts individuals worldwide. It is the second leading cause of cancer‐related deaths and is projected to increase by 60% by 2030 [1, 2]. The incidence and mortality rates of CRC vary considerably across different regions; Europe and Australia/New Zealand exhibit the highest incidence rates, while Eastern Europe has the highest mortality rates [2]. According to projections, the global incidence of CRC is anticipated to rise to 3.2 million new cases annually by 2040, representing a 63% increase [3]. Additionally, the number of deaths attributed to this disease is expected to reach 1.6 million per year, signifying a 73% rise [4].

Most cases of CRC occur in those over the age of 50 [5]. Several risk factors for CRC have been identified by studies, including dietary patterns, smoking, heavy alcohol consumption, physical inactivity, obesity, genetic, and epigenetic factors [6]. However, the precise mechanisms responsible for the development and progression of CRC remain unknown [7, 8]. CRC evolves over time, similar to other types of cancer, due to a series of epigenetic changes that primarily impact the genetic material. This process transforms normal colonic mucosa into malignant tumors. The transition typically begins with changes within polyps, particularly adenomas [9].

As a subclass of non‐coding RNAs (ncRNAs), long non‐coding RNAs (lncRNAs) have been discovered to play a significant role in numerous cellular functions, including the regulation of DNA, RNA, and proteins [10]. In recent years, lncRNAs have garnered significant interest due to their regulatory roles in gene expression across various biological processes [10, 11] including tumorigenesis and cancer progression [10, 12]. Defined as RNA molecules greater than 200 nucleotides in length, lncRNAs can act as either positive or negative regulators of gene expression [12, 13]. They perform significant functions such as serving as molecular sponges for microRNAs (miRNAs) or acting as both cis‐acting and trans‐acting regulators in chromatin interactions [14].

LncRNAs can be classified into several categories, including enhancer lncRNAs, intronic lncRNAs, antisense lncRNAs, intergenic lncRNAs, sense lncRNAs, circular lncRNAs, and bidirectional transcript [11]. They play a part in regulating tumor occurrence and growth, and changes in their expression levels can indicate different stages of the disease. Research indicates that specific lncRNAs are markedly upregulated in CRC tissues, contributing to tumor growth and metastasis [15]. Their expression levels often alter in tumors, indicating potential roles as diagnostic and prognostic markers and therapeutic targets [16].

Several studies report that particular lncRNAs, such as CRC‐associated transcript (CCAT), H19, lung adenocarcinoma transcript 1 (MALAT‐1), and HOTAIR, are frequently overexpressed in tumor tissues compared to normal tissues. Patients exhibiting high expression levels of these lncRNAs often have poorer prognoses [17]. Conversely, some lncRNAs function as tumor suppressors by inhibiting processes like cancer cell growth, invasion, and metastasis. For instance, Linc02023 suppresses CRC tumorigenesis by stabilizing PTEN [18, 19], while LINC01133 inhibits epithelial‐mesenchymal transition (EMT) and metastasis by modulating SRSF6 [20]. Additionally, reduced expression of lncRNA SATB2‐AS1 in CRC tissues correlates with tumor staging and poor patient outcomes [21].

In addition to sporadic cancer, various miRNAs have been identified as significant players in colitis‐associated cancer (CAC) [22]. For example, specific miRNAs such as miR‐331‐3p and hsa‐let‐7d‐5p have been implicated in the inflammatory pathways and tumorigenesis associated with colitis. These miRNAs are crucial in regulating inflammation, apoptosis, and cancer cell proliferation genes, positioning them as potential therapeutic targets [23].

Among emerging lncRNAs, EVADR has been identified as specifically expressed in tumors of glandular origin, including adenocarcinomas of the colon, rectum, lung, pancreas, and stomach. Moderate to high levels of EVADR expression are detected in 25%–53% of these tumors, and its expression is correlated with decreased patient survival [24, 25]. EVADR is located on chromosome 6q13, and its expression is notably low in non‐glandular tumor sites (under 10% detection in patients). Additionally, *Fusobacterium nucleatum* ( *F. nucleatum* ) has been implicated in the progression and metastasis of CRC by promoting tumor cell migration and invasion. Recent studies indicate that *F. nucleatum* upregulates the expression of the EVADR, which further enhances the metastatic potential of CRC cells [26, 27]. Another lncRNA of interest, LUESCC (NR_110801), located on chromosome 17q24.1, is upregulated in esophageal squamous cell carcinoma (ESCC) compared to adjacent normal tissues [28]. Reducing the expression of LUESCC led to decreased cell proliferation, colony formation, migration, and invasion in vitro, as well as inhibited tumor growth in vivo [28].

This case–control study aims to evaluate the expression levels of lncRNAs EVADR and LUESCC in colorectal tumor tissues and investigate their association with CRC risk. By elucidating the roles of these lncRNAs, we hope to contribute valuable insights for potential diagnostic and therapeutic strategies in the context of CRC.

## 2 Methods

### 2.1 Ethics Statement

The research was carried out in compliance with the Declaration of Helsinki and was approved by the Ethics Committee of Shahid Beheshti University of Medical Sciences (SBMU) under the code IR.SBMU.MSP.REC.1399.886. Written consent was acquired from all participants.

### 2.2 Methods and Materials

#### 2.2.1 Selection and Expression Analysis of lncRNAs in CRC

The expression of the lncRNAs LUESCC and EVADR was investigated using the UALCAN (http://ualcan.path.uab.edu/) database [29] to assess their levels in CRC. UALCAN provides comprehensive cancer transcriptome data from The Cancer Genome Atlas (TCGA) (https://www.cancer.gov/ccg/research/genome‐sequencing/tcga) [30] enabling the comparison of lncRNA expression in CRC samples versus normal tissue samples.

#### 2.2.2 Prediction of Potential miRNA Interactions

Based on the reference RNA sequences of the lncRNAs LUESCC and EVADR, potential miRNA interactions were predicted using the miRDB (http://mirdb.org) [31] and TargetScan (https://www.targetscan.org/vert_80/) databases [32]. These databases offer reliable tools for identifying miRNA binding sites and predicting miRNA targets.

#### 2.2.3 Construction of Interaction Networks

Interaction networks between the predicted miRNAs and lncRNAs were constructed using Cytoscape 3.10.3, a bioinformatics software platform for visualizing molecular interaction networks. This step helped in visualizing and better understanding the potential regulatory relationships between lncRNAs and miRNAs.

#### 2.2.4 Study Population

We procured 50 FFPE colorectal tumor tissue samples from individuals with sporadic CRC who underwent surgical excision at the Pathology Department of Imam Hossein Hospital in Tehran, Iran. The diagnosis of CRC followed the National Comprehensive Cancer Network (NCCN) guidelines [33], confirmed through clinical examination, colonoscopy, and histopathological analysis. The control group comprised tumor margin tissues from different individuals, confirmed to be free of neoplasia. All samples were fixed in buffered formalin before paraffin embedding. Epidemiological data—including age, sex, occupation, literacy, marital status, ethnicity, cancer stage and grade, family history, and other variables—were collected through structured interviews with all participants. CRC diagnoses were confirmed via clinical examination, colonoscopy, and histopathological analysis of biopsies. Patients with prior chemotherapy or radiation therapy were excluded, and none had a history of hereditary or malignant diseases.

#### 2.2.5 RNA Extraction and cDNA Synthesis

The extraction of RNA from paraffin‐embedded tissue was carried out using the Quick‐DNA/RNA FFPE Kit from ZymoResearch. The quality and concentration of the RNA were assessed based on the absorbance at 260/230 nm and 260/280 nm using the Thermo Scientific NanoDrop 2000. Subsequently, the RNA was preserved at −80°C until cDNA synthesis. For cDNA synthesis, 2 μg of total RNA was utilized along with random hexamer primers and the Yekta Tajhiz cDNA synthesis kit (Cat. no: YTA4500; Yekta Tajhiz, Iran).

#### 2.2.6 Quantitative Real‐Time PCR (qRT‐PCR)

Performing QRT‐PCR involved using 2 μL of cDNA in a 15 μL reaction volume. The qPCR reaction mix contained 10 μL of SYBR Green Real‐time PCR Master Mix Kit (Cat.no: YT2551; Yekta Tajhiz, Iran) and 0.5 μL of 100 nM of each forward and reverse primer (Table 1) for lncRNAs. Rotor‐Gene Q (Corbett Research, Sydney, AU) was used for quantitative RT‐PCR (Table 2). U6 was utilized as the housekeeping gene for data normalization. The Pfaffl method was applied for reporting fold changes.

**TABLE 1:** qRT‐PCR thermal cycles.

Gene	Forward primer	Thermal cycles		
		Hold	Cycle (*n* = 40)	Melt
EVADR	5′ CTGGTGCTTTGGCCTCATCT 3′ 5′ AGGACATTCAGTCTTCGGCG 3′	95°C 10 min	95°C 10s 60°C 30 s 72°C 25 s	72°C–95°C 5 min
LUESCC	5′ AAAGGTTTCCAAGCTGCGTC 3′ 5′ GGACCCTCTGGCTTTGGATT 3′			

**TABLE 2:** Demographic variables and clinical characteristics of the patients.

Group variable	Frequency (percent)
Gender	
Male	34 (78%)
Female	16 (22%)
Fecal occult blood	
Positive	14 (28%)
Negative	36 (72%)
Family history of cancer	
Positive	8 (16%)
Negative	42 (84%)
Tumor location	
Ascending colon	12 (24%)
Rectum	13 (26%)
Descending colon	12 (24%)
Sigmoid	4 (14%)
Cecum	9 (18%)
Tumor differentiation^1^	
Well	24 (48%)
Intermediated	12 (24%)
Poor	14 (28%)
Metastasis	
No	44 (88%)
Yes	6 (12%)

^1^ Tumor differentiation groups refer to classifications based on how much tumor cells resemble normal cells, with “well” differentiation indicating cells that closely resemble normal tissue, while “poor” differentiation indicates more abnormal cells.

### 2.3 Statistical Analysis

The relative expression analysis was conducted using the REST 2009 software (Qiagen, Hilden, Germany). Graph Pad Prism 8.0 was utilized for statistical analyses of the expression data. Normality of the data was assessed using the Shapiro–Wilk test. The rate of change of expression data was obtained using the *t*‐test. A significance level of two‐tailed *p* value < 0.05 was observed.

## 3 Results

A collective of 50 CRC patients (34 men and 16 women) who had an average age of 61.16 ± 10.77 years were included in this research study. The demographic variables and clinical characteristics of the patients are outlined in Table 2.

### 3.1 Gene Expression Data

**FIGURE 1:**
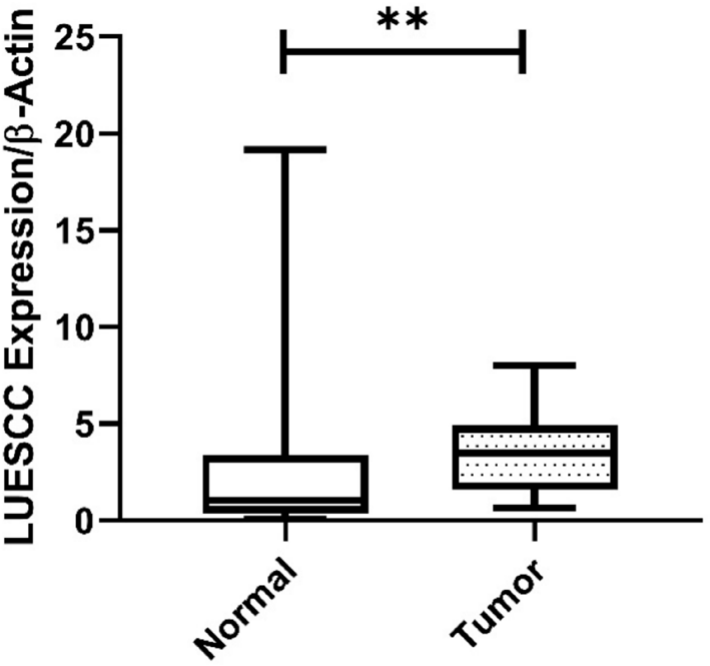
Gene expression data of LUESCC lncRNA using box plots. The relative expression levels of LUESCC lncRNA are compared between CRC patients (case group) and normal tissues (control group). The analysis revealed a significant increase in expression, with an average fold change of 3.52 (*p* < 0.001), determined using a *t*‐test. This figure illustrates the distinct differences in LUESCC expression levels, highlighting its potential as a diagnostic biomarker in CRC. CRC, colorectal cancer; lncRNA, long non‐coding RNA. ** *p* < 0.01.

**FIGURE 2:**
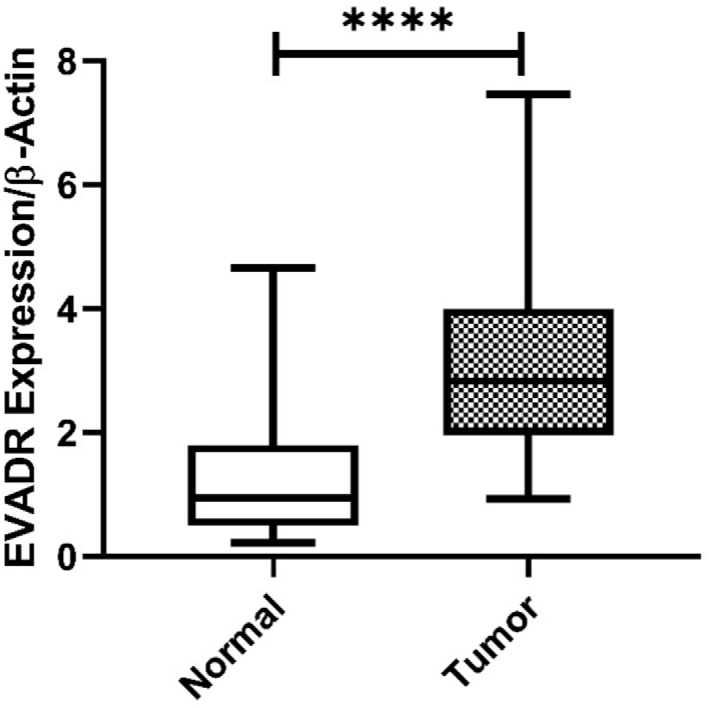
Gene expression data of EVADR lncRNA using box plots. This figure illustrates the relative expression levels of EVADR lncRNA in CRC patients compared to control groups. The data indicate a significant increase in EVADR expression, with an average fold‐change of 3.08 (*p* < 0.001), as determined by a *t*‐test. This suggests that EVADR lncRNA may play a role in CRC pathology. CRC, colorectal cancer; lncRNA, long non‐coding RNA. **** *p* < 0.0001.

**FIGURE 3:**
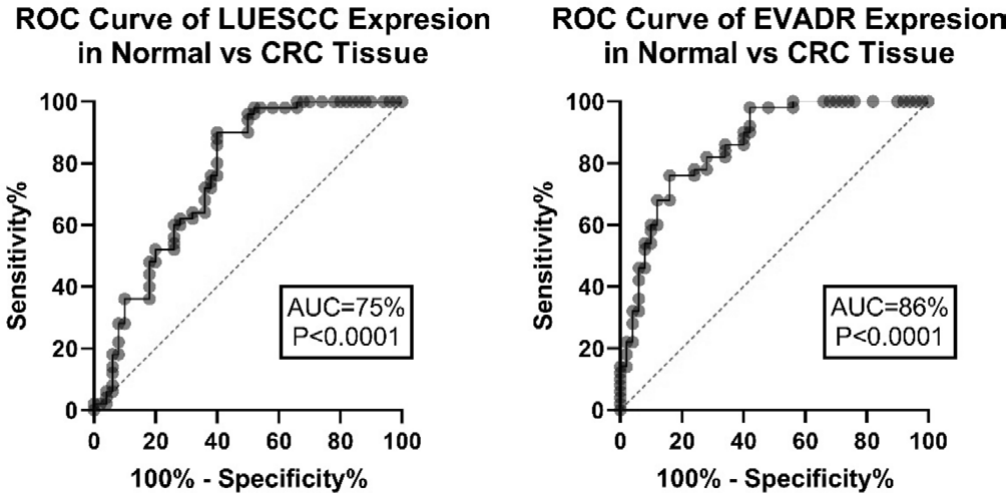
ROC curve analysis demonstrating the diagnostic potential of LUESCC and EVADR lncRNAs in CRC tumors. The AUC for LUESCC expression is 75% (*p* < 0.0001), indicating its ability to distinguish CRC from normal tissues. For EVADR, the AUC is 86% (*p* < 0.0001), further supporting its role as a potential biomarker for CRC diagnosis. The ROC curve illustrates sensitivity versus 1‐specificity, providing a visual representation of the diagnostic accuracy of these lncRNAs. AUC, area under curve; CRC, colorectal cancer; lncRNA, long non‐coding RNA; ROC, receiver operating characteristic.

The findings indicated a significant increase in the expression of LUESCC lncRNA in CRC patients, with an average fold‐change of 3.52 (*p* < 0.001) (Figure 1). Moreover, the expression of EVADR lncRNA was notably elevated in CRC patients, with an average fold‐change of 3.08 (*p* < 0.001), as depicted in Figure 2. ROC curve analysis demonstrated an AUC of 75% (*p* < 0.0001) for LUESCC expression and 86% (*p* < 0.0001) for EVADR in CRC tumors (Figure 3).

**FIGURE 4:**
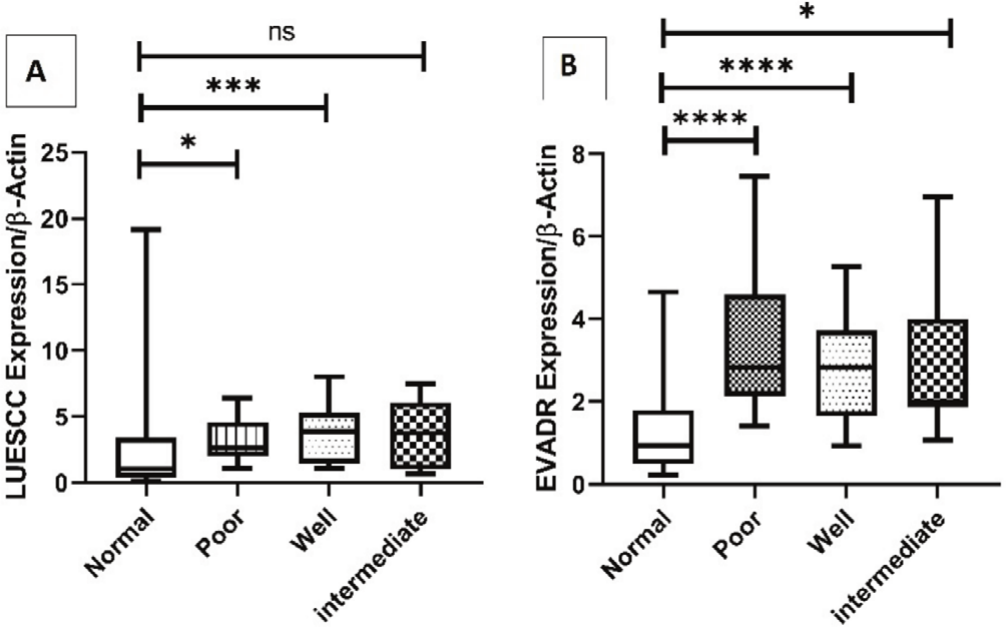
Expression levels of LUESCC (A) and EVADR (B) lncRNAs in CRC tissues compared to normal tissues. The analysis shows a significant increase in LUESCC expression in well‐differentiated (*p* = 0.0007) and poorly differentiated (*p* = 0.03) CRC groups. Additionally, EVADR expression is significantly elevated in well (*p* < 0.0001), poorly (*p* < 0.0001), and intermediate (*p* = 0.006) differentiation groups compared to normal tissues. Statistical significance was determined using the *t*‐test. lncRNA, long non‐coding RNA; CRC, colorectal cancer. * *p* < 0.05; *** *p* < 0.001; **** *p* < 0.0001.

Further analysis revealed that the expression of LUESCC in well (*p* = 0.0007) and poor (*p* = 0.03) differentiation groups in comparison with Normal tissues was significantly increased. Also, the expression of EVADR in well (*p* < 0.0001), poor (*p* < 0.0001), and intermediate (*p* = 0.006) differentiation groups in comparison with Normal tissues was significantly increased (Figure 4).

**FIGURE 5:**
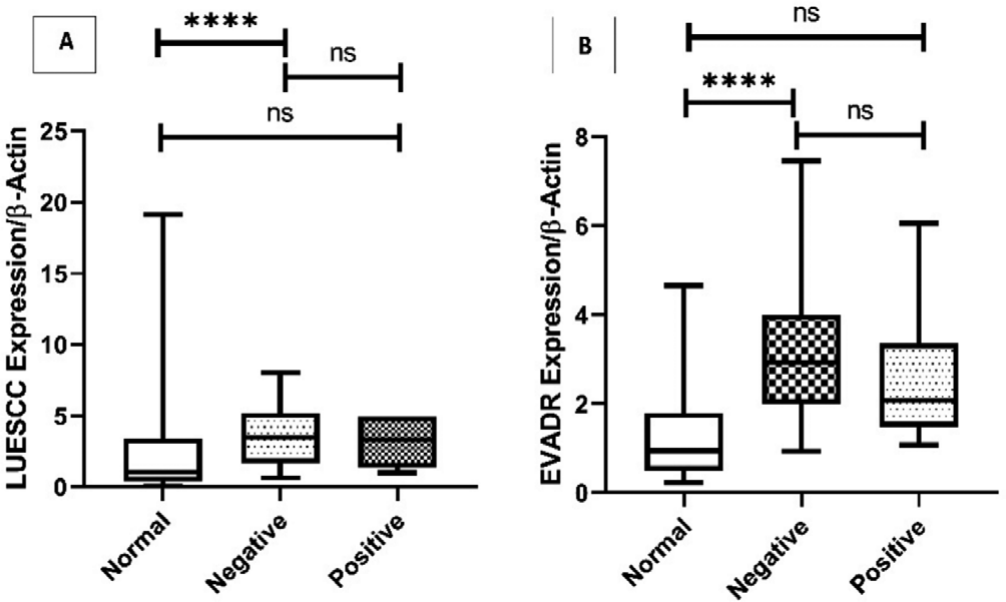
Expression levels of LUESCC and EVADR lncRNAs in CRC tissues based on metastasis status. The analysis shows a significant increase in the expression of LUESCC and EVADR in the negative metastasis group compared to normal tissues. In contrast, there were no significant changes observed in the positive metastasis group. Statistical significance was determined using the *t*‐test, with *p* values reported as follows: Positive metastasis (LUESCC: *p* = 0.04; EVADR: *p* < 0.0001) and Negative metastasis (LUESCC: *p* < 0.0001; EVADR: *p* < 0.0001). CRC, colorectal cancer; lncRNA, long non‐coding RNA. **** *p* < 0.0001.

On the other hand, the expression of LUESCC and EVADR was significantly increased in the negative metastasis group in comparison with normal tissues, but there were no significant changes in the positive metastasis group (Figure 5).

**FIGURE 6:**
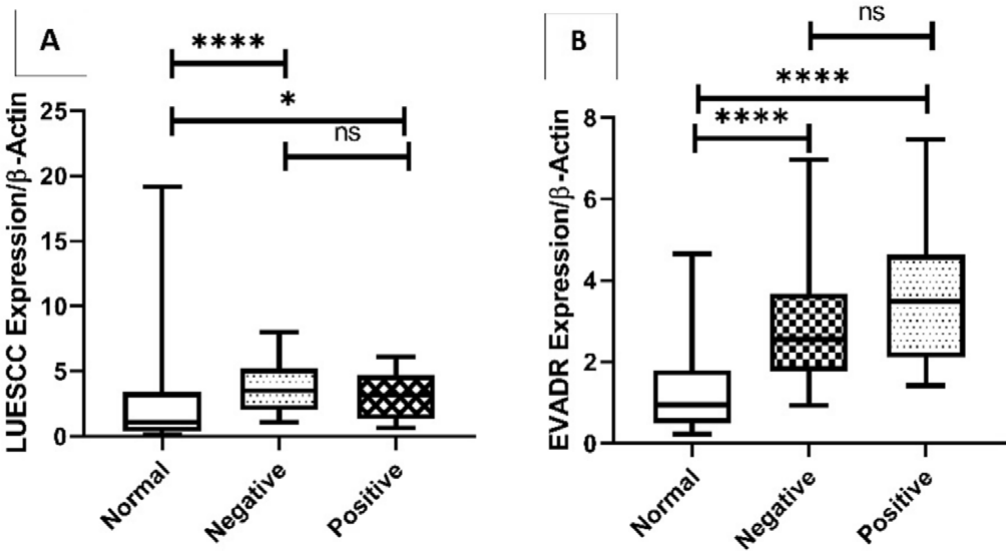
Expression levels of LUESCC and EVADR lncRNAs in relation to Fecal Occult Blood (FOB) results. The analysis shows a significant increase in the expression of both lncRNAs in positive FOB cases (LUESCC: *p* = 0.04; EVADR: *p* < 0.0001) and negative FOB cases (LUESCC: *p* < 0.0001; EVADR: *p* < 0.0001) compared to normal tissues. Statistical significance was determined using the *t*‐test. lncRNA, long non‐coding RNA. * *p* < 0.05; **** *p* < 0.0001.

In terms of fecal occult blood, there was a significant increase in expression of LUESCC and EVADR in Positive (*p* = 0.04 and *p* < 0.0001) and Negative (*p* < 0.0001 and *p* < 0.0001) groups in comparison with normal tissues (Figure 6). No significant change was observed between different age groups, gender, and family history.

### 3.2 Insilco Analysis of Target miRNAs

**FIGURE 7:**
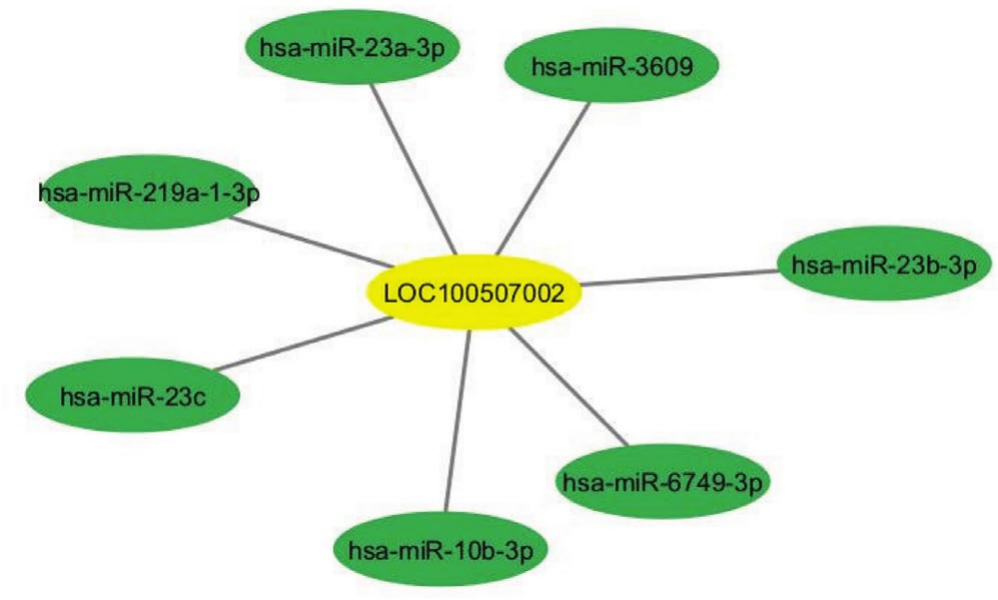
Network of LUESCC lncRNA and its predicted miRNA targets. This network was constructed using Cytoscape 3.10.3 and illustrates the potential regulatory interactions between LUESCC and various miRNAs implicated in CRC. CRC, colorectal cancer; lncRNA, long non‐coding RNA; miRNA, microRNA.

**FIGURE 8:**
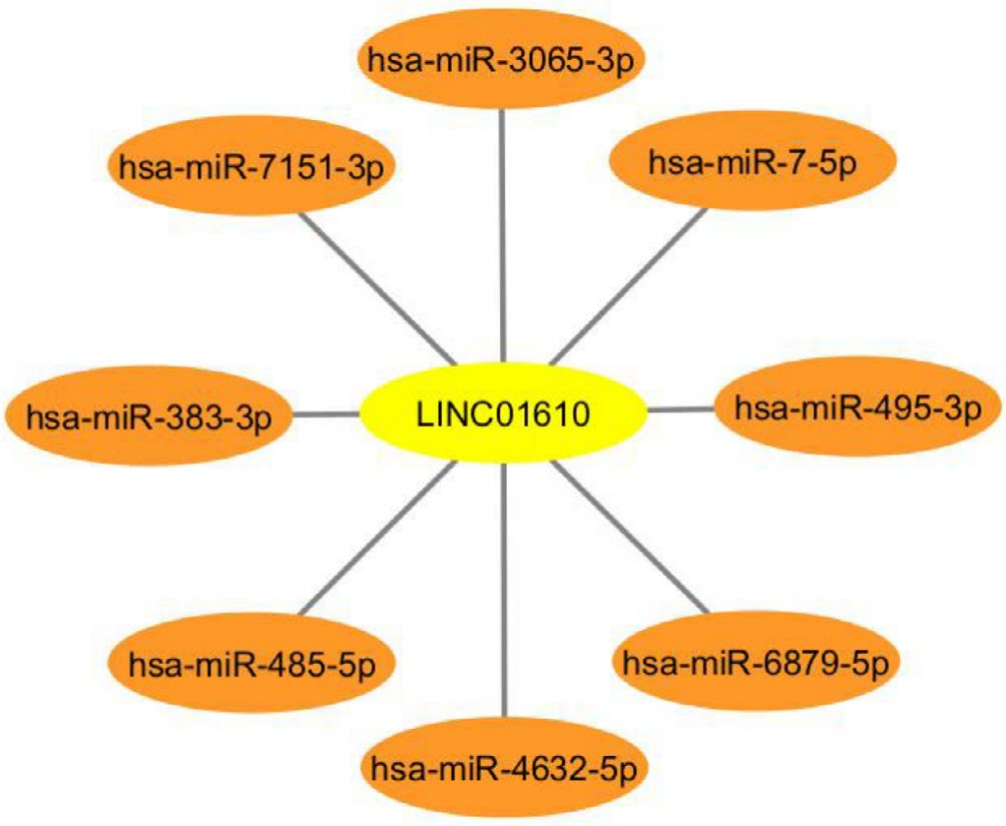
Network of EVADR lncRNA and its predicted miRNA targets. This network was constructed using Cytoscape 3.10.3 and illustrates the potential regulatory interactions between LUESCC and various miRNAs implicated in CRC. CRC, colorectal cancer; lncRNA, long non‐coding RNA; miRNA, microRNA.

The expression levels of the predicted miRNAs were further analyzed using the UALCAN database to determine their expression in CRC samples compared to normal samples. Additionally, data from the TCGA‐COAD (The Cancer Genome Atlas—Colon Adenocarcinoma) dataset were utilized to compare miRNA expression in cancerous and normal tissues (Tables 3 and 4). The Network of LUESCC and EVADR with their predicted miRNA targets were shown in Figures 7 and 8, respectively. This comparative analysis provided insights into the significance of these miRNAs in CRC.

**TABLE 3:** Predicted miRNAs targeted by LOC100507002 (LncRNA LUESCC).

Target detail	Target rank	Target score	miRNA name	Expression of miRNA in colorectal cancer (COAD)
LOC100507002	4	82	hsa‐miR‐6749‐3p	Down
LOC100507002	5	80	hsa‐miR‐10b‐3p	Up
LOC100507002	9	72	hsa‐miR‐23c	Down
LOC100507002	10	72	hsa‐miR‐23b‐3p	Down
LOC100507002	11	72	hsa‐miR‐23a‐3p	Up
LOC100507002	12	69	hsa‐miR‐219a‐1‐3p	Down
LOC100507002	18	51	hsa‐miR‐3609	Down

*Note:* Lists the miRNAs that are predicted to interact with the LUESCC lncRNA based on bioinformatics analyses.

**TABLE 4:** Predicted miRNAs targeted by LINC01610 (LncRNA EVADR).

Target detail	Target rank	Target score	miRNA name	Expression of miRNA in colorectal cancer (COAD)
LINC01610	2	91	hsa‐miR‐495‐3p	Down
LINC01610	3	89	hsa‐miR‐3065‐3p	Up
LINC01610	4	79	hsa‐miR‐7151‐3p	Down
LINC01610	6	75	hsa‐miR‐6879‐5p	Down
LINC01610	8	75	hsa‐miR‐4632‐5p	Down
LINC01610	10	75	hsa‐miR‐7‐5p	Down
LINC01610	14	71	hsa‐miR‐485‐5p	Down
LINC01610	15	71	hsa‐miR‐383‐3p	Down

*Note:* Lists the miRNAs that are predicted to interact with the EVADR lncRNA based on bioinformatics analyses.

## 4 Discussion

CRC is the most prevalent gastrointestinal cancer and a leading cause of cancer‐related deaths worldwide, linked to various genetic factors [34]. lncRNAs, previously thought to be non‐functional, are now recognized for their significant roles in cancer development by regulating gene expression through multiple mechanisms. Their dysregulation has been particularly noted in CRC, highlighting their importance in cancer biology [35]. Several recent studies have emphasized the important role of lncRNAs in controlling the biological processes of CRC. For instance, altered expression of lncRNA‐NEAT1 has been shown to impact cell proliferation, invasion, and migration both in vivo and in vitro [36]. Additionally, lncRNA‐SNHG15 has been identified as a regulator of downstream genes such as MYC, NRAS, BAG3, and ERBB3, all of which are closely associated with cancer progression [37, 38].

Several lncRNAs including CCAT2, Gata6, SNHG11, RPPH1, LINC01106, and SURC have been found to be significantly upregulated in CRC compared to normal colorectal tissues. For instance, lncRNA‐SNHG11 has been shown to interact with HIF‐1α and promote its expression, thereby contributing to the invasion and metastasis of CRC cells [39]. Similarly, the overexpression of lncRNA‐RPPH1 in CRC tissues has been linked to the promotion of epithelial‐EMT mechanisms [40]. Furthermore, LINC01106 has been found to trigger the activation of Gli family factors, which in turn contributes to the growth and maintenance of CRC [41]. Additionally, lncRNA‐APC1 has been identified as a regulator of CRC progression through noncanonical Wnt signaling [42]. Notably, high expression of lncRNA‐SURC has been associated with reduced disease‐free survival and overall survival in CRC patients, with SURC implicated in stimulating the expression of CCND2 by suppressing miR‐185‐5p expression in CRC cells [43].

The present study results offer valuable insights into the expression patterns of LUESCC and EVADR lncRNAs in CRC patients. The findings show a significant upregulation of both LUESCC and EVADR lncRNAs in CRC patients compared to normal tissues, with notable fold‐change values and statistical significance. The ROC curve analysis further underscores the potential diagnostic value of these lncRNAs, as indicated by high AUC values that demonstrate their ability to differentiate between CRC tumors and normal tissues. Additionally, the differential expression of LUESCC and EVADR lncRNAs in different differentiation groups suggests a potential association between these lncRNAs and CRC tumor grade. The significant increase in expression observed in well, poor, and intermediate differentiation groups compared to normal tissues underscores the potential role of these lncRNAs in CRC progression and aggressiveness.

This study also revealed distinct expression patterns of LUESCC and EVADR lncRNAs in relation to metastasis and fecal occult blood. While both lncRNAs were significantly upregulated in the negative metastasis group, no significant changes were observed in the positive metastasis group. In a study by Song‐tao Xue et al., it was found that LUESCC is primarily localized in the cytoplasm of ESCC cells, indicating its potential to influence post‐transcriptional regulation of mRNA targets through miRNA sponging. The study showed that by sponging miR‐6785‐5p, the lncRNA LUESCC regulates NRSN2 expression, thus facilitating the advancement of ESCC. Elevated levels of LUESCC are correlated with gender, deep invasion, lymph node metastasis, and poor prognosis in ESCC patients [28]. Moreover, the increased expression of LUESCC in cancerous tissues is particularly significant in light of the observed inverse relationship between LUESCC and miR‐219a‐1 expression levels. Xu et al. indicate that miR‐219a‐1 is downregulated in CRC and functions as a tumor suppressor by inhibiting cancer cell proliferation, invasion, and migration through targeting MEMO1 [44]. Given that lncRNA1 expression is upregulated while miR‐219a‐1 is downregulated in CRC, consistent with our bioinformatic results, it is plausible that LUESCC may play a role in the disease's progression, potentially through interaction or regulation involving miR‐219a‐1. Therefore, the upregulation of LUESCC in CRC tissues underscores its potential as a biomarker for diagnosis or prognosis, reflecting the underlying molecular alterations associated with the malignancy.

Xiaoxue Lu and colleagues found that *F. nucleatum* promotes the metastasis of CRC cells to the liver and lungs, as demonstrated in mouse models. They discovered that *F. nucleatum* increases EVADR expression, enhancing CRC cell metastasis through Y‐box binding protein 1 (YBX1), which facilitates the translation of factors linked to epithelial‐EMT. Our study identified different EVADR expression levels in human samples between metastatic and non‐metastatic groups, contrasting with the findings in the mouse model [27]. In a study by Yari and colleagues, it was revealed that EVADR ceRNA transcript variants enhance the WNT and PI3K signaling pathways in SW480 and HCT116 CRC cells by sponging miR‐7 and miR‐29b. Overexpression of EVADR resulted in increased cell cycle progression, viability, and migration, along with a reduced early/late apoptotic rate and a lower Bax/Bcl2 ratio in CRC cells [45]. It seems that EVADR and LUESCC are more involved in the initiation and progression of colon cancer than in the invasive stages.

In many cases, lncRNAs exert their functions by interacting with miRNAs, and in silico analyses have identified various miRNA targets for both lncRNAs. Our bioinformatics results suggest that lncRNA EVADR may serve as a significant biomarker for CRC due to its potential interactions with downregulated miRNAs such as miR‐495‐3p, miR‐7‐5p, and miR‐383‐3p. Specifically, miR‐495‐3p acts as a tumor suppressor in colorectal tumors by negatively regulating key genes involved in cell cycle progression, including TGFβR1, TGFβR2, SMAD4, and BUB1 [46]. Similarly, miR‐7‐5p, also downregulated in CRC tissues and cell lines, inhibits cell proliferation and migration by targeting Krüppel‐like factor 4 (KLF4) [47]. Furthermore, miR‐383‐3p, which is downregulated in CRC tissues, functions as a tumor suppressor by targeting pro‐tumor genes and is associated with tumor size, metastasis, and tumor stage [48]. The concurrent upregulation of lncRNA EVADR and the downregulation of these miRNAs in CRC suggests that EVADR may play a crucial role in the progression of the disease, potentially through its interactions with these miRNAs. Therefore, the increased level of EVADR in CRC tissues indicates its potential as a biomarker for the diagnosis or prognosis of the disease, indicating significant molecular changes associated with the cancer. Conducting future studies to investigate possible lncRNA‐miRNA interactions can provide us with more information.

This study presents several strengths that contribute to our understanding of CRC. Firstly, it is one of the first investigations to evaluate the expression patterns of lncRNAs LUESCC and EVADR specifically in an Iranian patient population, highlighting potential ethnic and regional differences in CRC biology. Secondly, our robust methodology, including comprehensive statistical analyses and bioinformatics approaches, enhances the reliability of our findings. The significant upregulation of LUESCC and EVADR in CRC tissues compared to normal samples, along with high AUC values from ROC curve analysis, underscores their potential as diagnostic biomarkers. Furthermore, the observed associations between lncRNA expression and tumor differentiation suggest their roles in CRC progression and aggressiveness, providing valuable insights into potential therapeutic targets.

Overall, the results provide valuable insights into the diagnostic and prognostic capabilities of LUESCC and EVADR lncRNAs in CRC. The lack of significant associations with age, gender, and family history also suggests that these lncRNAs may serve as independent biomarkers for CRC diagnosis and monitoring.

## 5 Conclusion

Our study demonstrates a significant upregulation of both LUESCC and EVADR lncRNAs in CRC patient tissues, with notable fold‐change values and statistical significance. These findings suggest that LUESCC and EVADR have potential as diagnostic biomarkers in CRC tumors. Importantly, the lack of significant associations with age, gender, and family history indicates that these lncRNAs may serve as independent biomarkers for CRC diagnosis and monitoring.

## 6 Limitations

While this study provides valuable insights into the expression of LUESCC and EVADR lncRNAs in CRC, several limitations should be acknowledged. The sample size of 50 cases may limit the generalizability of the findings; therefore, larger studies with more diverse populations are necessary for confirmation. We have added a statement in Section 6 acknowledging that while we included tumor margin tissues confirmed to be free of neoplasia as a control group, incorporating a third group with samples from healthy colon tissue would have strengthened our comparative analysis. This limitation has been noted for consideration when interpreting our findings. Additionally, the absence of functional assays, such as CRISPR‐Cas9 knockouts or overexpression models, limits our understanding of the specific roles these lncRNAs play in tumor biology. Addressing these limitations in future research is crucial for elucidating the clinical significance of these findings.

## 7 Recommendations and Future Perspectives

Based on our findings, we recommend validating LUESCC and EVADR as CRC biomarkers in larger, diverse cohorts. Functional studies, such as CRISPR‐Cas9 knockouts or overexpression models, are needed to elucidate their roles in tumor biology. Investigating interactions with specific microRNAs will clarify their regulatory networks. Longitudinal studies could assess their prognostic value in clinical settings. Exploring therapeutic targeting of these lncRNAs and their interactions with tumor microenvironment components, like immune cells or stromal factors, may lead to new CRC treatment strategies.

## Author Contributions

Mozhgan Ahmadzadeh and Kamal Shahamiri contributed equally to this work as co‐first authors. They were responsible for conceptualization, methodology, data analysis, and drafting the manuscript. Mohammad Raeisi and Negar Jafari contributed to data collection and initial interpretation. Shaghayegh Kamian and Mahsa Ejlalidiz participated in statistical analysis and data visualization. Niloufar Sadat Kalaki supervised the study, critically reviewed the manuscript, and approved the final version. All authors read and approved the final manuscript.

## Ethics Statement

This study was approved by the ethics committee in the school of medicine, Shahid Beheshti University of medical sciences with research ethics approval ID IR.SBMU.RETECH.REC.1399.886. All patients signed an informed consent form before surgery and agreed to their tissue samples being used in the research project. All ethical requirements were observed during sample collection.

## Consent

The authors have nothing to report.

## Conflicts of Interest

The authors declare no conflicts of interest.

## Data Availability

Research data are not shared.
